# Whole-Body Insulin Sensitivity Rather than Body-Mass-Index Determines Fasting and Post-Glucose-Load Growth Hormone Concentrations

**DOI:** 10.1371/journal.pone.0115184

**Published:** 2014-12-17

**Authors:** Christian-Heinz Anderwald, Andrea Tura, Alois Gessl, Sabina Smajis, Christian Bieglmayer, Rodrig Marculescu, Anton Luger, Giovanni Pacini, Michael Krebs

**Affiliations:** 1 Division of Endocrinology and Metabolism, Department of Internal Medicine III, Medical University of Vienna, Vienna, Austria; 2 Metabolic Unit, Institute of Biomedical Engineering, National Research Council (ISIB-CNR), Padua, Italy; 3 Mariahilf Community Pharmacy, Arnoldstein, Austria; 4 Medical Direction, Specialized Hospital Complex Agathenhof, Micheldorf, Austria; 5 Clinical Institute of Medical and Chemical Laboratory Diagnostics, Medical University of Vienna, Vienna, Austria; Heidelberg University, Germany

## Abstract

**Background:**

Obese, non-acromegalic persons show lower growth hormone (GH) concentrations at fasting and reduced GH nadir during an oral glucose tolerance test (OGTT). However, this finding has never been studied with regard to whole-body insulin-sensitivity as a possible regulator.

**Methods:**

In this retrospective analysis, non-acromegalic (NonACRO, n = 161) and acromegalic (ACRO, n = 35), non-diabetic subjects were subdivided into insulin-sensitive (IS) and –resistant (IR) groups according to the Clamp-like Index (CLIX)-threshold of 5 mg·kg^−1^·min^−1^ from the OGTT.

**Results:**

Non-acromegalic IS (CLIX: 8.8±0.4 mg·kg^−1^·min^−1^) persons with similar age and sex distribution, but lower (p<0.001) body-mass-index (BMI = 25±0 kg/m^2^, 84% females, 56±1 years) had 59% and 70%, respectively, higher (p<0.03) fasting GH and OGTT GH area under the curve concentrations than IR (CLIX: 3.5±0.1 mg·kg^−1^·min^−1^, p<0.001) subjects (BMI = 29±1 kg/m^2^, 73% females, 58±1 years). When comparing on average overweight non-acromegalic IS and IR with similar anthropometry (IS: BMI: 27±0 kg/m^2^, 82% females, 58±2 years; IR: BMI: 27±0 kg/m^2^, 71% females, 60±1 years), but different CLIX (IS: 8.7±0.9 vs. IR: 3.8±0.1 mg·kg^−1^·min^−1^, p<0.001), the results remained almost the same. In addition, when adjusted for OGTT-mediated glucose rise, GH fall was less pronounced in IR. In contrast, in acromegalic subjects, no difference was found between IS and IR patients with regard to fasting and post-glucose-load GH concentrations.

**Conclusions:**

Circulating GH concentrations at fasting and during the OGTT are lower in non-acromegalic insulin-resistant subjects. This study seems the first to demonstrate that insulin sensitivity rather than body-mass modulates fasting and post-glucose-load GH concentrations in non-diabetic non–acromegalic subjects.

## Introduction

The standardized, 75 g-oral glucose tolerance test (OGTT) is a widespread tool to predominantly diagnose disorders of glucose metabolism, such as glucose intolerance and (gestational) diabetes mellitus, but is also applied for confirmation or exclusion of clinical suspicion of acromegaly (ACRO) [1]–[3]. The OGTT GH decline below the threshold of 1 ng/mL is currently thought to exclude ACRO presence, in addition to a concentration of insulin–like growth factor-1 within the age-specific reference range [1]–[4].

Several studies were conducted to elucidate the mechanism, by which plasma GH declines during an OGTT in non–acromegalic (NonACRO) persons. GH secretion is orchestrated by hypothalamic peptides and systemic hormones: GH synthesis and secretion are stimulated and amplified, respectively, by GH-releasing-hormone (GHRH) and the GH secretagogue ghrelin [1], [5]. Following an oral glucose load, GH concentrations decline rapidly and markedly, most likely because of inhibition of GHRH and/or stimulation of somatostatin release [5]. Moreover, it has been known for long that GH concentrations at fasting differ between normal-weight and obese persons [6]–[8]. Numerous research groups, who have dealt with physiological differences in OGTT GH, reported that baseline concentrations, nadir and/or rebound of circulating GH during and/or after an OGTT were significantly associated with body-mass-index (BMI), fasting insulin concentrations, visceral fat mass, trunk fat, high-sensitivity C–reactive protein, and mitochondrial function, as well as leptin and adiponectin concentrations [5]–[15]. Interestingly, all these parameters underlie –from an endocrinologist's view– the influence of whole-body insulin-sensitivity [16], [17]. Of note, the role of insulin sensitivity has never been investigated in this context in detail in NonACRO.

Thus, we hypothesized that insulin-resistant and insulin-sensitive persons display different GH responses to an oral glucose load; this should occur rather in NonACRO, but not in ACRO, because ACRO patients have inadequately and insuppressibly elevated GH secretion, which defines the disease [1].

To this end, we aimed to analyze the impact of insulin resistance on GH decline during an OGTT by using our large database conducted over more than a decade, containing comprehensive OGTT outcome in non-acromegalic and acromegalic subjects. We employed advanced mathematical modeling to determine insulin-sensitivity-indexes for distinction between insulin-sensitive and -resistant persons, who were grouped according to the glucose infusion rate (equivalent) threshold of insulin resistance of <5 mg·kg^−1^·min^−1^, as defined by others and ourselves [18]–[20]. Thereafter, in order to prove our hypothesis, we compared NonACRO subjects with similar body mass, but different degrees of insulin sensitivity, with regard to OGTT GH (decline). Finally, we studied insulin–sensitive and insulin–resistant ACRO patients, in whom -owing to insuppressible GH release– no strong effect of whole–body insulin–sensitivity on OGTT GH decrease was anticipated.

## Materials and Methods

### Study participants

The data of those subjects, who had been admitted between Jan 2001 and Sep 2011 to the Endocrine Outpatients Ward of our department, were electronically composed by computer–assisted collection. General exclusion criteria were: diabetes mellitus, age >75 years, impaired kidney function, pronounced liver injury including cirrhosis, Cushing's syndrome, pituitary surgery, adrenal cortex carcinoma, status post adrenalectomy, phaeochromocytoma, insulinoma, hyperprolactinemia, and aldosteronism; and additionally, for the NonACRO, pituitary tumors and acromegaly, with regard to the current guidelines [1]–[4]. We included all subjects with fasting GH of at least 0.3 ng/mL, who had undergone an OGTT. The data compositions and the analyses were approved by the local ethics committee of the Vienna Medical University (#1970/2012). Because of the retrospective analysis, no consent was obtained in any form. The local ethics committee approved this procedure including the waiver of a (written) consent.

### Oral glucose tolerance test

Participants were instructed to arrive at our Endocrine Outpatients Ward in fasting condition, meaning an at least 10-hour period without consumption of food or beverages except for water. Blood was drawn after insertion of a catheter (Vasofix; Braun, Melsungen, Germany) into one antecubital vein at fasting, and 60, 90, and 120 min after drinking a solution consisting of 75 g glucose (Gluco-Drink75; Roche Diagnostics, Vienna, Austria) for determination of plasma glucose and subsequent analyses of hormones [19], [21], [22]. Samples were centrifuged and then either immediately analyzed or frozen at −80°C.

### Group formations

Following the strictly defined inclusion and exclusion criteria listed above, of the 453 subjects in the entire database, 161 met the criteria for eligibility as non-acromegalic subjects (NonACRO), and 35 those for acromegalic patients (ACRO). As elaborated by others and ourselves [18]–[20], the cut-off-level of decreased insulin sensitivity was set at <5 mg·kg^−1^·min^−1^. Insulin sensitivity was determined by the Clamp–like Index [19] that not only tightly correlates with glucose infusion rates in the hyperinsulinemic-isoglycemic clamp–test, but also yields values of insulin sensitivity very close to those of the clamp-test. Therewith, we obtained an insulin–sensitive (IS) and insulin–resistant (IR) group of both NonACRO and ACRO. Finally, we performed a selection of the overweight IS- and IR-NonACRO subjects with similar BMI, but different insulin sensitivity, thereby termed IS-NonACRO-sBMI (n = 22) and IR-NonACRO-sBMI (n = 42), respectively, to allow comparison of OGTT GH decline in persons with comparable BMI, but different insulin sensitivity.

### Measurements

Parameters of clinical chemistry as well as circulating concentrations of glucose, insulin, and C-peptide were measured at the Department of Medical and Chemical Laboratory Diagnostics (www.kimcl.at), as described [19], [21]–[23].

Three different methods had to be used for GH measurement: Until Jan 2005, GH was determined with a time-resolved fluoroimmunoassay kit (Delfia, Wallac Oy, Turku, Finland) using an automated AutoDelfia system [6]. Because of a technical breakdown, this analyzer had to be replaced and method comparison studies were carried out with the Nichols Advantage hGH assay (Nichols Institute Diagnostika GmbH, Bad Vilbel, Germany) and IMMULITE 2000 hGH assay (Diagnostic Products Corp., Biermann GmbH, Bad Nauheim, Germany). *Passing-Bablok* regression analyses resulted in Advantage  = 1.24·DELFIA-0.004 (r = 0.88, n = 77) and IMMULITE  = 1.40·DELFIA +0.16 (r = 0.99, n = 73). The average bias ±2 SD to DELFIA was +0.30±0.67 for Advantage and +0.55±0.99 for IMMULITE. Because of the smaller bias, the laboratory decided to switch to Nichols Advantage hGH. This assay was used from Feb 2006 onward, but unexpectedly all Nichols products were removed from the market in Dec 2006. Thus, finally the IMMULITE 2000 hGH [7] assay was used.

The influence and potential bias of the 3 different GH measurement methods were investigated by comparison of the examination dates of all the participants' groups: Neither the examination dates of IS-NonACRO compared with IR-NonACRO (p = 0.977), nor those of IS-NonACRO-sBMI compared with IR-NonACRO-sBMI (p = 0.352), nor those of IS-ACRO compared with IR-ACRO (p = 0.413) showed any statistically significant time-dependent difference in hormone and metabolite analyses, which seems to rule out any impact of the GH detection method on the inter-group results.

### Calculations

Measures of insulin sensitivity, such as the Clamp–like Index (CLIX) for whole-body insulin-sensitivity, the oral glucose insulin sensitivity (OGIS) index for glucose clearance, the homeostatic model assessment of insulin resistance (HOMA-IR) and quantitative insulin sensitivity check index (QUICKI), the latter both suited for assessing hepatic insulin resistance [24], [25], as well as parameters of beta-cell function, such as the basal insulin secretion rate and the Insulinogenic Index (IGI) of 0–120 min were obtained as described in details elsewhere [19], [22], [23], [25]–[28]. The product of insulin sensitivity with an index of post-hepatic insulin appearance (sometimes termed Disposition Index) and that with C-peptide derived beta-cell function (sometimes termed Adaptation Index) provides figures of the capacity of the beta-cell to adapt its secretion to the changes in insulin resistance [23]. A rather novel insulin secretion index derived from OGTT C-peptide concentrations, called WHole-Ogtt-SHape-index-C-Peptide (WHOSH_CP), was determined as described elsewhere [23], [29]. Total and dynamic (Δ) areas under the curve (AUCs) were calculated by using the trapezoidal rule [23], [25]. Hepatic insulin extraction (as percentage of the secreted hormone) and fasting endogenous glucose production (EGP) were calculated as described elsewhere [23], [25].

### Statistical analyses

All data are given as means ± SEM. Before further analysis, the distribution of the variables was tested by visual examination for marked non-Normality and/or the *Kolmogorov-Smirnov* test, yielding that every variable was normally distributed. Comparisons between two, or more than two groups, were done by using two-tailed unpaired *Student*'s t-tests, or ANOVA with *post hoc* least significant difference (LSD) tests, respectively. Linear methods were used for correlation analyses using *Pearson*'s correlation coefficient *r*. Multiple linear regression analyses, based on the data of NonACRO and ACRO of all participants and the (sub-)groups, were applied twice with total AUC of OGTT GH concentrations and fasting GH concentrations as dependent variables, in order to find possible predictors for baseline and AUC GH in all participants and within each group. Predictors (i.e. independent parameters) of baseline and AUC GH at a significance level of p<0.10 remained in the model, as described [20], [30]. The final model was verified by backward stepwise multiple linear regression analysis.

Differences were considered statistically significant at p-values ≤0.05. Statistical analyses were performed by using SPSS (SPSS Inc., Chicago, IL) computer software.

## Results

### Anthropometrical characteristics with baseline examination and lab values (Table 1)

Apart from higher body mass (i.e. BMI and body weight) in IR-NonACRO (each p<0.001 vs. IS-NonACRO), the three IR- and IS-groups did not differ among other anthropometrical characteristics (each p≥0.1). IS-NonACRO had 5 mmHg lower diastolic blood pressure and their serum triglyceride concentrations were reduced by 37%, whereas their HDL-cholesterol was 11% higher than in IR-NonACRO (each p<0.02). No other differences with regard to serum parameters of kidney and thyroid gland as well as lipids were found among the groups.

### OGTT

**Figure 1:**
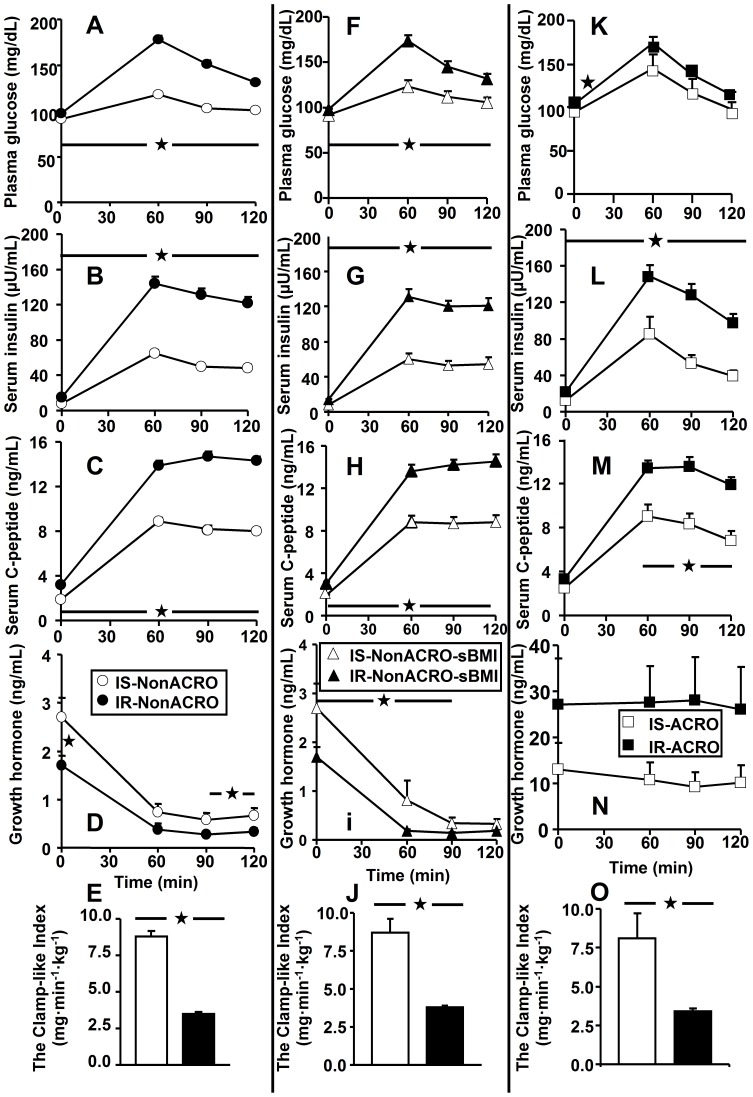
Results of the oral glucose tolerance test with time courses of concentration of plasma glucose, serum insulin serum C–peptide and growth hormone as well as the Clamp-like Index are given for insulin resistant (IR) and insulin sensitive (IS) subjects of the 3 groups. (**A–E**) non-acromegalic subjects (NonACRO: •, IR [n = 88]; o, IS [n = 73]), (**F–J**) non-acromegalic subjects with similar body mass index (NonACRO-sBMI: ▴, IR [n = 42]; Δ, IS [n = 22]), and (**K–O**) acromegalic patients (ACRO: ▪, IR [n = 23]; □, IS [n = 12]). Differences were analyzed by using the *Student*'s t–test: *, p<0.05.

The time courses of circulating glucose, insulin and C-peptide during the OGTT were markedly different between IS and IR in all three groups (Fig. 1A–C, F–H, K–M), all of which were well reflected by their respective total and dynamic AUCs (Table 1). Of note, IR- and IS-ACRO had similar OGTT glucose concentrations, as well as total and dynamic glucose AUCs. Fasting EGP was higher by 27% in IR-ACRO (p<0.009 vs. IS-ACRO, p<0.001 vs. IS-NonACRO and IR-NonACRO), whereas hepatic insulin extraction was lower (p<0.01 IS vs. IR) in all 3 IR subgroups, when compared to the respective IS subgroups (Table 1).

**Table 1:** Anthropometrical characteristics and baseline examination and laboratory values, oral glucose tolerance test (OGTT) results and areas under the curve (AUCs), estimates of insulin sensitivity and insulin secretion parameters and indexes in non-diabetic insulin-resistant (IR) and –sensitive (IS) subjects without acromegaly (NonACRO), without acromegaly and with similar body mass index ((NonACRO-sBMI), and acromegaly (ACRO).

	IR-NonACRO	IS-NonACRO	IR-NonACRO-sBMI	IS-NonACRO-sBMI	IR-ACRO	IS-ACRO
n	88	73	42	22	23	12
**Anthropometrical characteristics and baseline examination and lab values**						
Sex (% females)	73%	84%	71%	82%	52%	50%
Age (years)	58±1	56±1	60±1	58±2	49±3	46±4
Weight (kg)	81±2 $	70±2 $	74±1	75±2	81±4	80±5
Height (cm)	167±1	168±1	167±1	168±2	173±2	174±3
BMI (kg/m^2^)	29±1 $	25±0 $	27±0	27±0	27±1	26±1
Systolic blood pressure (mmHg)	145±2	138±3	148±4	150±7	137±5	135±3
Diastolic blood pressure (mmHg)	87±1 *	82±1 *	87±2	87±3	86±3	84±3
Creatinine (mg/dL)	0.88±0.02	0.9±0.02	0.91±0.03	0.93±0.03	0.77±0.03	0.98±0.18
Total cholesterol (mg/dL)	220±4	219±5	222±6	224±7	207±9	202±8
HDL cholesterol (mg/dL)	58±2 #	65±2 #	60±2	63±3	54±3	55±4
LDL cholesterol (mg/dL)	134±4	134±3	135±5	141±5	130±8	129±6
Triglycerides (mg/dL)	140±6 $	102±5 $	137±8 +	105±7 +	114±13	87±8
TSH (µU/mL)	2.0±0.1	2.3±0.3	2.1±0.2	2.9±1	1.2±0.2	1.7±0.4
**OGTT results and areas under the curve**						
Glucose 0 min (mg/dL)	97±1 $	91±1 $	98±1 §	92±2 §	103±2 ÷	94±3 ÷
Glucose 120 min (mg/dL)	131±3 $	101±2 $	132±5 §	106±5 §	114±4	98±8
Insulin 0 min (µU/mL)	15.2±1 $	8.3±0.4 $	13.6±1.2 §	7.8±0.6 §	21.2±2.5 ÷	12.3±2.2 ÷
Growth hormone 0 min (ng/mL)	1.7±0.2 *	2.7±0.4 *	1.7±0.2 +	2.7±0.5 +	27.1±10	13.1±5.7
Impaired fasting glucose (%)	39% #	16% #	43%	27%	61%	33%
Glucose intolerance (%)	40.9% $	2.7% $	42.9% §	4.5% §	8.70%	8.30%
Diabetes mellitus (%)	0%	0%	0%	0%	0%	0%
Total AUC of OGTT glucose (mg/dL x min)	17437±327 $	12683±250 $	17131±505 €	13258±541 €	16778±610	14319±1254
Total AUC of OGTT insulin (µU/mL x min)	12728±613 $	5377±275 $	11742±668 €	5335±461 €	12651±935 £	6354±932 £
Total AUC of OGTT C-peptide (ng/ml x min)	1379±34 $	821±25 $	1344±52 €	847±50 €	1288±66 £	833±89 £
Dynamic AUC of OGTT glucose (mg/dL x min)	5749±301 $	1720±231 $	5320±473 €	2251±507 €	4465±461	2999±1110
Dynamic AUC of OGTT insulin (µU/mL x min)	10904±535 $	4386±249 $	10110±605 €	4398±428 €	10113±853 £	4876±826 £
Dynamic AUC of OGTT C-peptide (ng/ml x min)	992±27 $	591±20 $	983±45 €	616±42 €	888±58 ¶	535±60 ¶
Total AUC of OGTT growth hormone (ng/mL x min)	83±11 #	141±18 #	66±7 §	132±27 §	3286±1059	1307±489
Dynamic AUC of OGTT growth hormone (ng/mL x min)	−126±19	−179±32	−136±19	−188±53	40±302	−270±211
Total AUC of growth hormone per total AUC of glucose (ng/mL x min per g/dL x min)	4.9±0.6 $	11.6±1.6 $	4.0±0.5 §	11.4±3.1 §	200±66	107±45
Fasting endogenous glucose production (mg/kg/min)	1.4±0.1	1.4±0.1	1.4±0.1	1.3±0.1	1.9±0.1 ¶	1.4±0.1 ¶
Hepatic insulin extraction (0–120 min OGTT) (%)	41±2 $	55±2 $	40±2 €	56±3 €	33±3 ¶	48±4 ¶
**Further estimates of insulin sensitivity**						
OGIS 120 min (ml/min/m^2^)	341±5 $	429±6 $	341±7 €	423±13 €	338±8 £	434±17 £
HOMA-IR	3.7±0.2 $	1.9±0.1 $	3.3±0.3 €	1.8±0.1 €	5.4±0.7 ÷	2.9±0.5 ÷
QUICKI	0.38±0 $	0.42±0 $	0.38±0.01 €	0.42±0.01 €	0.35±0.01 ¶	0.39±0.01 ¶
**Insulin secretion parameters and indexes**						
Basal insulin secretion rate (pmol/min)	256±12 $	144±6 $	228±12 €	147±9 €	282±27	208±32
Fasting beta-cell function (nmol/mmol), prehepatic	0.198±0.008 $	0.125±0.005 $	0.182±0.009 €	0.126±0.007 €	0.194±0.016	0.155±0.02
Fasting beta-cell function (pmol/mmol), posthepatic	19.5±1.3 $	11.3±0.6 $	17.2±1.5 §	10.6±0.8 §	25.6±2.9	16.4±3
Insulinogenic Index (total insulin AUC, 0–120 min) (pmol/mmol)	96±6 $	53±3 $	90±7 €	52±5 €	96±8 ¶	60±11 ¶
Adaptation Index (ml min^−1^m^−2^) x (nmol/mmol)	166±6	168±6	167±9	168±13	158±10	160±21
Disposition Index (ml min^−1^m^−2^) x (nmol/mmol)	31±2 $	22±1 $	30±2 +	22±2 +	32±2	27±6
WHOSH_CP	0.0061±0.0004 *	0.0048±0.0004 *	0.0055±0.0004	0.0049±0.0008	0.0063±0.0007	0.0057±0.0012

Differences were analyzed by using the *Student*'s t–test: *, p<0.05 IR-NonACRO vs. IS-NonACRO; #, p<0.01 IR-NonACRO vs. IS-NonACRO; $, p<0.001 IR-NonACRO vs. IS-NonACRO; +, p<0.05 IR-NonACRO-sBMI vs. IS-NonACRO-sBMI; §, p<0.01 IR-NonACRO-sBMI vs. IS-NonACRO-sBMI; €, p<0.001 IR-NonACRO-sBMI vs. IS-NonACRO-sBMI; ÷, p<0.05 IR-ACRO vs. IS-ACRO; ¶, p<0.01 IR-ACRO vs. IS-ACRO; £, p<0.001 IR-ACRO vs. IS-ACRO.

### Growth hormone before and during the OGTT (Table 1 & Fig. 1)

Fasting GH concentrations were 59% (each p<0.05 vs. IR) higher in IS subgroups of both NonACRO and NonACRO-sBMI, but similar between IR- and IS-ACRO. Except for only one time-point (60 min in NonACRO and 120 min in NonACRO-sBMI), non-acromegalic IS persons had elevated GH concentrations during the OGTT (each p<0.05), when compared to the corresponding IR subgroups (Fig. 1D+i). IS- and IR-ACRO showed comparable GH concentrations during the OGTT (Fig. 1N). Total AUC of GH during the OGTT was 66–100% greater in non-acromegalic IS (each p<0.006 vs. IR), whereas in ACRO, no difference was found between IR and IS (Table 1). Dynamic AUC of GH was similar between IR and IS in all 3 groups. In order to determine the suppressive effect of the glucose rise on GH concentrations during the OGTT, we calculated the AUC ratio of GH to glucose, which was lower in both groups of non-acromegalic IR (each p<0.003 vs. respective IS), but comparable between IR-ACRO and IS-ACRO (Table 1).

### Markers of insulin sensitivity (Table 1 & Fig. 1)

Not only whole-body insulin sensitivity (CLIX) (Fig. 1E+J+O), but also glucose clearance (OGIS) and hepatic insulin sensitivity (HOMA and QUICKI) (Table 1) were markedly different between IR and IS in all 3 groups (each p<0.02), thus indicating the clearly decreased insulin action in all IR groups.

### Insulin secretion (indexes) (Table 1)

Of the non-acromegalic subjects, the IS had lower basal secretion rates, as obvious from pre- and posthepatic beta-cell function, and reduced insulin-based (Insulinogenic and Disposition) Indexes during the OGTT (each p≤0.01), whereas the C-peptide-based Adaptation Index was similar in the OGTT. In the acromegalic patients, however, of all the insulin secretion parameters, only the Insulinogenic Index was different and higher by 60% in the patients with insulin resistance (p<0.01 vs. IS-ACRO).

### Correlation and regression analyses

CLIX and HOMA-IR were, respectively, negatively and positively, associated with BMI in all participants (r = −0.352, p<0.001, and r = 0.511, p<0.001), NonACRO (r = −0.373, p<0.001, and r = 0.610, p<0.001), and, in part borderline significantly, in ACRO (r = −0.306, p = 0.07, and r = 0.435, p = 0.009).

Thereafter, we chose the parameters of total GH AUC and fasting GH as major dependent variables for correlation and regression analyses. In the correlation analyses (Table 2), BMI and measures of both insulin sensitivity and insulin secretion were associated with both GH OGTT AUC and fasting GH. Importantly, in the ACRO, no associations of GH with BMI nor with CLIX and OGIS were found, but tight negative relationships with most insulin secretion indexes, in particular with regard to GH AUC during the OGTT (each r≥0.4, each p<0.02).

**Table 2:** Correlation coefficients (*Pearson*–correlation moment products) and significance levels (p–values) of total of area under the curve (AUC) of growth hormone (GH) and basal growth hormone concentrations with anthropometric measures, parameters of the baseline clinical lab, OGTT outcomes, as fasting endogenous glucose production, hepatic insulin extraction, values of insulin sensitivity and indexes of insulin secretion in non- acromegalic (NonACRO) and acromegalic (ACRO) subjects separated.

	Total AUC of GH (ng/mL x min)		GH 0 min (ng/mL)	
	NonACRO	ACRO	NonACRO	ACRO
Sex (% females)	r = −0.042, p = 0.596	r = −0.191, p = 0.272	**r = −0.156, p = 0.049**	r = −0.206, p = 0.236
Age (years)	**r = −0.169, p = 0.032**	r = −0.189, p = 0.276	**r = −0.157, p = 0.047**	r = −0.214, p = 0.217
Weight (kg)	**r = −0.250, p = 0.001**	r = 0.174, p = 0.316	**r = −0.155, p = 0.05**	r = 0.167, p = 0.337
Height (cm)	r = 0.07, p = 0.375	r = 0.206, p = 0.235	**r = 0.168, p = 0.034**	r = 0.216, p = 0.214
BMI (kg/m^2^)	**r = −0.306, p<0.001**	r = 0.094, p = 0.589	**r = −0.247, p = 0.002**	r = 0.07, p = 0.688
Systolic blood pressure (mmHg)	r = −0.034, p = 0.676	r = 0.044, p = 0.812	r = 0.034, p = 0.678	r = 0.011, p = 0.952
Diastolic blood pressure (mmHg)	r = −0.129, p = 0.113	r = −0.102, p = 0.578	r = 0.032, p = 0.693	r = −0.178, p = 0.331
Creatinine (mg/dL)	r = 0.009, p = 0.911	r = 0.05, p = 0.775	r = 0.112, p = 0.155	r = 0.055, p = 0.752
Total cholesterol (mg/dL)	r = −0.097, p = 0.226	r = −0.421, p = 0.016	r = −0.124, p = 0.121	**r = −0.384, p = 0.03**
HDL cholesterol (mg/dL)	r = −0.013, p = 0.869	r = −0.293, p = 0.104	r = −0.002, p = 0.983	r = −0.311, p = 0.084
LDL cholesterol (mg/dL)	r = −0.048, p = 0.554	**r = −0.423, p = 0.016**	r = −0.083, p = 0.302	**r = −0.373, p = 0.035**
Triglycerides (mg/dL)	r = −0.199, p = 0.013	r = 0.152, p = 0.406	**r = −0.208, p = 0.009**	r = 0.156, p = 0.395
TSH (µU/mL)	**r = 0.244, p = 0.002**	r = 0.024, p = 0.893	r = 0.011, p = 0.891	r = 0.033, p = 0.85
Glucose 0 min (mg/dL)	**r = −0.233, p = 0.003**	r = 0.1, p = 0.566	**r = −0.178, p = 0.024**	r = 0.111, p = 0.526
Glucose 120 min (mg/dL)	r = −0.077, p = 0.331	r = −0.063, p = 0.72	r = −0.045, p = 0.573	r = −0.072, p = 0.679
Insulin 0 min (µU/mL)	**r = −0.244, p = 0.002**	**r = 0.586, p<0.001**	**r = −0.201, p = 0.011**	**r = 0.544, p = 0.001**
Growth hormone 0 min (ng/mL)	**r = 0.755, p<0.001**	**r = 0.972, p<0.001**	**-**	**-**
Total AUC of OGTT glucose (mg/dL x min)	**r = −0.161, p = 0.042**	r = 0.046, p = 0.794	r = −0.135, p = 0.088	r = 0.051, p = 0.77
Total AUC of OGTT insulin (µU/mL x min)	**r = −0.213, p = 0.007**	**r = 0.453, p = 0.006**	r = −0.122, p = 0.122	**r = 0.362, p = 0.033**
Total AUC of OGTT C-peptide (ng/ml x min)	**r = −0.243, p = 0.002**	**r = 0.415, p = 0.013**	r = −0.148, p = 0.061	r = 0.3, p = 0.08
Dynamic AUC of OGTT glucose (mg/dL x min)	r = −0.104, p = 0.189	r = 0.012, p = 0.945	r = −0.093, p = 0.241	r = 0.014, p = 0.935
Dynamic AUC of OGTT insulin (µU/mL x min)	**r = −0.195, p = 0.013**	**r = 0.334, p = 0.05**	r = −0.101, p = 0.202	r = 0.243, p = 0.16
Dynamic AUC of OGTT C-peptide (ng/ml x min)	r = −0.196, p = 0.013	r = 0.196, p = 0.258	r = −0.096, p = 0.227	r = 0.083, p = 0.634
Total AUC of OGTT growth hormone (ng/mL x min)	**-**	**-**	**r = 0.755, p<0.001**	**r = 0.972, p<0.001**
Dynamic AUC of OGTT growth hormone (ng/mL x min)	**r = −0.454, p<0.001**	**r = −0.372, p = 0.028**	**r = −0.927, p<0.001**	**r = −0.58, p<0.001**
Total AUC of growth hormone per total AUC of glucose (ng/mL x min per g/dL x min)	**r = 0.95, p<0.001**	**r = 0.984, p<0.001**	**r = 0.696, p<0.001**	**r = 0.948, p<0.001**
Dynamic AUC of growth hormone per total AUC of glucose (ng/mL x min per g/dL x min)	**r = −0.448, p<0.001**	r = −0.299, p = 0.081	**r = −0.903, p<0.001**	**r = −0.513, p = 0.002**
Fasting endogenous glucose production (mg/kg/min)	**r = −0.168, p = 0.033**	r = 0.141, p = 0.42	**r = −0.211, p = 0.007**	r = 0.138, p = 0.429
Hepatic insulin extraction (0–120 min OGTT) (%)	r = 0.136, p = 0.093	**r = −0.352, p = 0.038**	r = 0.087, p = 0.286	**r = −0.336, p = 0.048**
CLIX (mg/kg/min)	**r = 0.286, p<0.001**	r = −0.192, p = 0.269	**r = 0.156, p = 0.048**	r = −0.156, p = 0.371
OGIS 120 min (ml/min/m^2^)	**r = 0.278, p<0.001**	r = −0.152, p = 0.384	r = 0.112, p = 0.158	r = −0.072, p = 0.682
HOMA-IR	**r = −0.255, p = 0.001**	**r = 0.535, p = 0.001**	**r = −0.210, p = 0.008**	**r = 0.502, p = 0.002**
QUICKI	**r = 0.358, p<0.001**	**r = −0.446, p = 0.007**	**r = 0.329, p<0.001**	**r = −0.413, p = 0.014**
Basal insulin secretion rate (pmol/min)	**r = −0.246, p = 0.002**	**r = 0.613, p<0.001**	**r = −0.183, p = 0.02**	**r = 0.569, p<0.001**
Fasting beta-cell function (nmol/mmol), prehepatic	**r = −0.229, p = 0.003**	**r = 0.641, p<0.001**	**r = −0.179, p = 0.023**	**r = 0.573, p<0.001**
Fasting beta-cell function (pmol/mmol), posthepatic	**r = −0.224, p = 0.004**	**r = 0.617, p<0.001**	**r = −0.185, p = 0.019**	**r = 0.567, p<0.001**
Insulinogenic Index (total insulin AUC, 0–120 min) (pmol/mmol)	**r = −0.178, p = 0.024**	**r = 0.446, p = 0.007**	r = −0.092, p = 0.248	**r = 0.347, p = 0.041**
Adaptation Index (ml min^−1^m^−2^) x (nmol/mmol)	r = −0.045, p = 0.572	r = 0.299, p = 0.081	r = −0.042, p = 0.597	r = 0.238, p = 0.168
Disposition Index (ml min^−1^m^−2^) x (nmol/mmol)	r = −0.147, p = 0.063	**r = 0.401, p = 0.017**	r = −0.085, p = 0.285	**r = 0.354, p = 0.037**
WHOSH_CP	r = −0.153, p = 0.053	r = 0.245, p = 0.156	**r = −0.169, p = 0.032**	r = 0.227, p = 0.191

In the backward stepwise multiple linear regression analyses (Table 3), systolic blood pressure, sex, age, BMI and insulin sensitivity (i.e. CLIX) were included as predictors; with comprehensive analyses of the study participants, first combining all subjects, and then grouping them according to presence or absence of insulin resistance and/or acromegaly. With regard to total AUC of OGTT GH, predominantly age, BMI, and insulin sensitivity, but also once systolic blood pressure, were predictors; however, yielding CLIX as the most important predictor by far. With regard to fasting GH, sex, age, BMI, and insulin sensitivity were predictors, showing CLIX and sex to be the most frequent predictors in our second model.

**Table 3:** Multiple linear regression analyses with total area under the curve (AUC) of oral glucose tolerance test (OGTT) growth hormone and baseline ( = 0 min) growth hormone as dependent variables, and systolic blood pressure, sex, age, body mass index (BMI) and insulin sensitivity, determined by the Clamp–like Index (CLIX) as predictors, in all participants, all insulin resistant (IR) combined, all insulin sensitive (IS) combined, and non- acromegalic (NonACRO) subjects combined, with IR and IS subgroups, and all acromegalic (ACRO) combined, again with subgroups.

	Total AUC of OGTT growth hormone						Growth hormone 0 min					
		Systolic blood pressure	Sex (f = 1, m = 0)	Age	BMI	Insulin sensitivity (CLIX)		Systolic blood pressure	Sex (f = 1, m = 0)	Age	BMI	Insulin sensitivity (CLIX)
**All participants**												
*r*	0.334						0.363					
B±SE				−26.3±6.1	−25.2±15.1	−62±21.9			−2.9±1.4	−0.2±0.1	−0.2±0.1	−0.4±0.2
p-value				**<0.001**	0.097	**0.005**			**0.04**	**<0.001**	0.057	**0.02**
												
**All IR combined**												
*r*	0.456						0.533					
B±SE				−35.1±8.8	−57.8±21	−431.7±132.7			−4.3±1.6	−0.2±0.1	−0.4±0.1	−3.2±0.9
p-value				**<0.001**	**0.007**	**0.002**			**0.008**	**<0.001**	0.004	**<0.001**
												
All IS combined												
*r*	0.208						0.265					
B±SE				−13.5±7.2						−0.2±0.1		−0.4±0.3
p-value				0.063						**0.024**		0.159
												
**All NonACRO**												
*r*	0.363						0.298					
B±SE					−4.0±1.9	9.0±2.9			−1.1±0.4		−0.07±0.04	0.10±0.06
p-value					**0.031**	**0.002**			**0.019**		0.064	0.068
												
**IR-NonACRO**												
*r*	0.394						0					
B±SE		0.8±0.4		−1.5±0.9	−5.2±1.8	−19.7±11.9						
p-value		0.053		0.098	**0.005**	0.102						
												
**IS-NonACRO**												
*r*	0.288						0.318					
B±SE						12.7±5.1			−2.2±0.9		−0.2±0.1	
p-value						**0.016**			**0.025**		0.08	
												
**All ACRO**												
*r*	0.381						0					
B±SE				−37.9±22.9		−153.6±87.3						
p-value				0.108		0.089						
												
**IR-ACRO**												
*r*	0.514						0.755					
B±SE						−1061.9±406.1			−9.7±4.5	−0.3±0.1		−7.3±2.3
p-value						**0.017**			**0.045**	0.067		**0.006**
												
**IS-ACRO**												
*r*	0						0					
B±SE												
p-value												

The regression coefficient (*r*) with B± standard error of the mean (SE) and p-value is given for each outcome; in case the model did not yield satisfactory results, an *r*–value of 0 is listed.

## Discussion

This study dealt with the potential impact of whole-body insulin–sensitivity on circulating GH concentrations at fasting and during an oral glucose tolerance test in non-diabetic subjects, in which 161 non-acromegalic subjects and 35 acromegalic patients could be included. The participants were first grouped according to presence or absence of acromegaly, and then further divided into insulin-sensitive and -resistant subgroups.

The main findings of this study are:

1. Insulin-resistant, non-acromegalic subjects have lower fasting and post-glucose-load GH concentrations, whereas no difference was therein found between insulin-sensitive and insulin-resistant acromegalic patients.
2. Our findings in non-acromegalic subjects could be confirmed in that, when comparing on average overweight persons with comparable body-mass-index, sex distribution, and age, but different insulin sensitivity degree, insulin-resistant subjects again showed reduced fasting and OGTT GH concentrations.
3. Fasting and total AUC of GH were negatively related to body mass index, body weight, and parameters of insulin sensitivity, but only in non-acromegalic subjects, whereas ACRO showed tight positive relationships of fasting GH and GH AUC with insulin secretion parameters.
4. The regression analyses yielded insulin sensitivity to be the most frequent predictor of GH at fasting and during the OGTT.
5. Insulin-resistant acromegalic patients showed higher fasting endogenous glucose production than did non-acromegalic subjects and insulin-sensitive acromegalic patients, whose EGP was comparable to that of non-acromegalics.

### Study outcome significance

Previous studies in non-acromegalic humans were focused to investigate fasting levels and/or regulation of circulating GH concentrations with regard to anthropometric measures and/or mere indicators of insulin resistance, such as hyperinsulinemia, adiponectin concentrations and subclinical inflammation markers [5]–[15]. To the best of our knowledge, this is the first study to demonstrate whole-body insulin sensitivity to be a regulator of circulating GH concentrations, both in fasting condition and after high caloric nutrient ingestion. In our opinion, this novel finding may add important information to endocrinologists for defining clearer guidelines with regard to the likelihood of acromegaly appearance in people with rather unspecific and slowly progressing symptoms of acromegaly such as broadened extremities, widened and thickened fingers, lips and soft tissue, as well as widened noses, bulges in forehead, and superimposed facial lines [3].

### Growth hormone and insulin sensitivity interaction

Insulin resistance not only becomes evident as reduced insulin–dependent glucose utilization, but also as higher fasting and/or less suppressible EGP, and higher lipid availability, such as circulating free fatty acids (FFA) and/or triglycerides [26]. Elevated GH concentrations contribute to the development of insulin resistance [31], since experimental infusion of GH induced a sustained increase in FFA and subsequently insulin resistance, measured by the clamp–test [32]. On the other hand, GH replacement in adults with severe growth hormone deficiency and obese type-2 diabetic patients resulted in increased whole-body insulin-sensitivity [11], [33].

### Possible mechanisms

It seems generally accepted that GH suppression during an OGTT predominantly occurs because of the rise in plasma glucose concentrations [6]. A good explanation for the GH fall in the postprandial state seems the “feast and famine cycle”: Therein, the rise in GH during fasting or starvation leads to increased circulating FFA output, whereas in the postprandial period, the release of both –GH and FFA– is diminished [31]. This “feast and famine cycle” points at a mutual interaction between presence of energy-rich substrates in the blood, such as FFA, glucose and/or amino-acids, and GH secretion. On the other hand, elevated circulating energy-rich substrates go along with and are regarded as a sign of insulin resistance, since in insulin-resistant persons, circulating FFA are higher at fasting, and less suppressed by insulin [20], [26], [34]; and a glucose load results in a more pronounced rise of plasma glucose concentrations, as also visible in the IR-NonACRO. As experimental GH infusion induces FFA release and reduces glucose utilization by the muscle [32], a mutual relationship between energy-rich circulating substrates with GH secretion seems likely. However - to the best of our knowledge - it has never been investigated in non-acromegalic non-diabetic humans, whether a mutual interaction between insulin sensitivity and GH secretion exists. This bidirectional interaction hypothesis could explain the lower fasting and OGTT GH concentrations in insulin-resistant non-diabetic subjects, who have higher glucose and FFA concentrations before and during an OGTT [20], [26]. Of note, Cornford *et al*. showed that in the absence of change in body weight, after 3 days of overeating that resulted in hyperinsulinemia -and probably insulin resistance- mean 24-h plasma GH concentrations declined nearly by 80% [9].

In order to assess the efficacy of the energy–rich substrate glucose in blood to suppress GH release, we calculated the ratio of total AUCs of GH to glucose. Surprisingly, we found that this ratio of total AUCs of GH to glucose was more than 50% lower in insulin-resistant subjects, indicating a less suppressive action of circulating glucose to central GH release. This diminished response to post-glucose-load plasma glucose elevations was not only seen in IS-NonACRO and IR–NonACRO, but also in the subgroups IS-NonACRO-sBMI and IR-NonACRO-sBMI, who were entirely matched for major anthropometric characteristics, including body mass.

In general, body mass and whole-body insulin-sensitivity are negatively related with each other [25], [26]. Thus, it appears difficult to distinguish between the effects of one or the other. However, in order to give more meaningful results, we selected two subgroups of insulin-sensitive and insulin-resistant non-acromegalic subjects with similar anthropometry, in whom the results of higher fasting and post-glucose-load GH concentrations and greater suppressibility of GH release by glucose in insulin-sensitive subjects persisted. Furthermore, since the correlation analyses revealed body mass and insulin sensitivity as more or less equal determinants of GH concentrations, we performed regression analyses, in which we found insulin sensitivity to be a more frequent predictor of fasting and post-glucose-load GH concentrations than the BMI.

### Clinical implications

Our results might have additional impact on the judgment of insulin sensitivity in subjects under suspicion of acromegaly. In the light of the discussion to lower the cut-off-value for the diagnosis of active acromegaly, e.g. to 0.4 ng/mL, it cannot be ruled out that in very highly insulin sensitive persons, high GH concentrations at fasting would not fall below the currently defined threshold during the OGTT. Thus, they might be –falsely- diagnosed as acromegalics.

Another finding with regard to clinical application is the fact that insulin–resistant, but not –sensitive, acromegalic patients showed elevated EGP, which is in general seen only following type-2 diabetes manifestation [21]. Thus, acromegalic patients should be screened for the presence of insulin resistance, either using OGTT indexes, or other insulin resistance indicators, as precisely described by Tam *et al.* [18], since acromegalics with insulin resistance seem most prone for developing type-2 diabetes.

### Limitations

Because of the retrospective study design from clinical routine, interesting circulating metabolites, such as FFA, which are not routine parameters, were not measured. Another drawback is the use of three different GH measurement methods, which do not easily allow for any further conclusions on GH thresholds in healthy people. However, as precisely described in the Methods section, there was no time–dependent difference between the examination dates among the studied subgroups. From this, biases due to GH measurement methods on the key findings of this study seem very unlikely. In addition, we regret that waist circumference is not provided. It would be interesting to determine whether insulin sensitivity is a determinant of GH levels even after adjusting for waist.

### Conclusions

Growth hormone at fasting and during OGTT is lower in non–acromegalic subjects with low insulin sensitivity. In addition, when adjusted for OGTT-mediated glucose rise, GH fall is less pronounced in insulin resistance. It therefore appears that insulin sensitivity rather than body mass affects GH concentrations at fasting and during an OGTT in non-diabetic non-acromegalic subjects.
